# Operating for Cataract under the Influence of Mercury

**Published:** 1847-11

**Authors:** 


*Operating for Cataract under the Influence of Mercury.*—M. Tavignot addressed a note to the Academy, stating that he looked upon mercurial salivation as a means of preventing many of the evils of inflammation after the operation for cataract. He seems to have imbibed this notion from the general observation of the influence of mercury in acute inflammation of the iris and cornea, and from considering that such a condition of those parts of the eye is what is to be feared after operating. He has put this idea to the test, having operated on three patients, who were just beginning to be affected by mercury, and in whom, too, there were some complications. He effected a perfect cure in from three to five weeks, having had no ills resulting from inflammation. The mercury is continued two or three days after operating, combined with extract of opium, so that the salivation induced may be most severe, just at the time when the ordinary precursors of iritis, or of corneitis, make their appearance—that s, about the third or sixth day after the operation.—*Ibid.*

